# Effects of Histotripsy on Local Tumor Progression in an *in vivo* Orthotopic Rodent Liver Tumor Model

**DOI:** 10.34133/2020/9830304

**Published:** 2020-11-25

**Authors:** Tejaswi Worlikar, Mishal Mendiratta-Lala, Eli Vlaisavljevich, Ryan Hubbard, Jiaqi Shi, Timothy L. Hall, Clifford S. Cho, Fred T. Lee, Joan Greve, Zhen Xu

**Affiliations:** 1 Department of Biomedical Engineering, University of Michigan, Ann Arbor, Michigan 48109, USA; 2 Division of Abdominal Radiology, University of Michigan, Ann Arbor, Michigan 48109, USA; 3 Department of Biomedical Engineering and Mechanics, Virginia Polytechnic Institute and State University, Blacksburg, Virginia 24061, USA; 4 Department of Pathology, University of Michigan, Ann Arbor, Michigan 48109, USA; 5 Department of Surgery, University of Michigan, Ann Arbor, Michigan 48109, USA; 6 Department of Surgery, VA Ann Arbor Healthcare System, Ann Arbor, Michigan 48105, USA; 7 Department of Radiology, University of Wisconsin, Madison, Wisconsin 53705, USA

## Abstract

**Objective and Impact Statement.:**

This is the first longitudinal study investigating the effects of histotripsy on local tumor progression in an *in vivo* orthotopic, immunocompetent rat hepatocellular carcinoma (HCC) model.

**Introduction.:**

Histotripsy is the first noninvasive, nonionizing, nonthermal, mechanical ablation technique using ultrasound to generate acoustic cavitation to liquefy the target tissue into acellular debris with millimeter accuracy. Previously, histotripsy has demonstrated *in vivo* ablation of noncancerous liver tissue.

**Methods.:**

N1-S1 HCC tumors were generated in the livers of immunocompetent rats (*n* = 6, control; *n* = 15, treatment). Real-time ultrasound-guided histotripsy was applied to ablate either 100% tumor volume + up to 2mm margin (*n* = 9, complete treatment) or 50-75% tumor volume (*n* = 6, partial treatment) by delivering 1-2 cycle histotripsy pulses at 100 Hz PRF (pulse repetition frequency) with *p* – ≥30MPa using a custom 1MHz transducer. Rats were monitored weekly using MRI (magnetic resonance imaging) for 3 months or until tumors reached ~25mm.

**Results.:**

MRI revealed effective post-histotripsy reduction of tumor burden with near-complete resorption of the ablated tumor in 14/15 (93.3%) treated rats. Histopathology showed <5mm shrunken, non-tumoral, fibrous tissue at the treatment site at 3 months. Rats with increased tumor burden (3/6 control and 1 partial treatment) were euthanized early by 2-4 weeks. In 3 other controls, histology revealed fibrous tissue at original tumor site at 3 months. There was no evidence of histotripsy-induced off-target tissue injury.

**Conclusion.:**

Complete and partial histotripsy ablation resulted in effective tumor removal for 14/15 rats, with no evidence of local tumor progression or recurrence.

## 1. Introduction

Liver cancer is a leading cause of cancer-related deaths worldwide and among the top ten in the US [1, 2], Hepatocellular carcinoma (HCC) is the most common primary liver cancer, accounting for >65% of all liver cancers [3]. In the United States, the incidence of HCC has more than tripled since 1980 [4]. Five-year survival rates for liver cancer in the US are reported at only 18%, the second lowest among all cancer types [1]. HCC is the most frequently seen in cirrhotic livers, often diagnosed at advanced stages of the disease when patients have become symptomatic and demonstrate concomitant liver dysfunction. However, with improved HCC screening of the cirrhotic population, tumors are identified at an earlier stage, and patients have a variety of locoregional treatment options.

The management of HCC involves a complex decision-making process, taking into account not only the tumor extent (i.e., size, location, number, vascular invasion, or extrahepatic involvement) and patient comorbidities but also the severity of liver dysfunction, as most treatments for HCC can exacerbate underlying liver disease [5]. Currently, curative treatment options for HCC include liver transplantation, surgical resection, or thermal ablation [6]. However, approximately 80% of HCC patients are not surgical candidates. Patients with HCC eligible for liver transplant require an extensive evaluation and a mandatory waiting period resulting in lengthening wait times for a donor organ [7, 8]. Even then, almost 70% and 20% of patients undergoing resection and transplantation, respectively, develop recurrent HCC [9, 10]. Locoregional therapies, such as transcatheter arterial chemoembolization (TACE), transarterial radioembolization (TARE), stereotactic body radiotherapy (SBRT), and percutaneous image-guided ablations including radiofrequency ablation (RFA), microwave ablation (MWA), and cryoablation, are commonly used for early or intermediate stage treatment [11, 12]. High-intensity focused ultrasound (HIFU) guided by real-time magnetic resonance imaging (MRI) or ultrasound is a noninvasive thermal ablation method that deposits continuous ultrasound waves or long ultrasound bursts to generate sudden, localized heat to destroy focal tumor by coagulative necrosis. Locoregional therapy has demonstrated improved disease-free and overall survival in patients who cannot undergo resection and is used as a bridge to transplantation or for downstaging borderline-eligible patients for transplant [13]. However, each form of locoregional therapy has different limitations based on tumor location and extent of disease, as well as morbidity based on the degree of underlying liver dysfunction secondary to cirrhosis [4, 14-16].

Histotripsy is the first noninvasive, nonionizing, nonthermal, mechanical ablation technique that employs acoustic cavitation to destroy target tissue with millimeter accuracy [17, 18]. Using microsecond duration, low duty cycle (on-time/total time <0.1%) and high-pressure (*p* – ≥ 15 MPa) ultrasound pulses applied from outside the body and focused inside the target tissue, cavitation microbubbles are generated from endogenous nanometer gas pockets in the target tissue with millimeter accuracy [17-19]. The rapid expansion and collapse of cavitation bubbles result in a high strain that disrupts the cellular structure in the target region to form a liquid acellular homogenate [18, 19]. The low duty cycle of the histotripsy pulses (ultrasound on-time/total treatment time≪1%) prevents undesirable thermal effects and may allow homogeneous ablations in highly vascular organs such as the liver [20]. In addition, the structural integrity of large blood vessels, nerves, and bile ducts can be preserved inside the treatment region due to the higher mechanical strength and resistance of collagen-based tissue to histotripsy-induced damage in comparison to noncollagenous tissues such as parenchymal organs and tumors [21-24]. Though histotripsy ablation can be visualized on both ultrasound and MRI [25, 26], we are currently using ultrasound imaging for real-time treatment guidance and MRI for posttreatment monitoring.

In previous studies, we have demonstrated that histotripsy can noninvasively break down noncancerous liver tissue into liquefied homogenate with no intact cellular structure in a human-scale porcine model and rodent model [20, 23, 27, 28], with the ablated homogenate completely absorbed by the body within a month. In an immunocompromised subcutaneous HCC murine model, histotripsy was able to ablate the target tumor which was resorbed over time, resulting in an effective reduction of local tumor progression and improvement in survival outcomes [29]. However, due to lack of treatment margin, untreated viable residual tumor at the boundary eventually led to progression. In this longitudinal study, the effect of partial and complete histotripsy tumor ablation on local tumor progression was investigated in an orthotopic immunocompetent rodent HCC model for the first time.

## 2. Results

### 2.1. Clinical Monitoring.

All tumor inoculation procedures and histotripsy procedures were well tolerated with no minor or major complications or deaths.

### 2.2. Local Tumor Response to Histotripsy.

**Figure 1:**
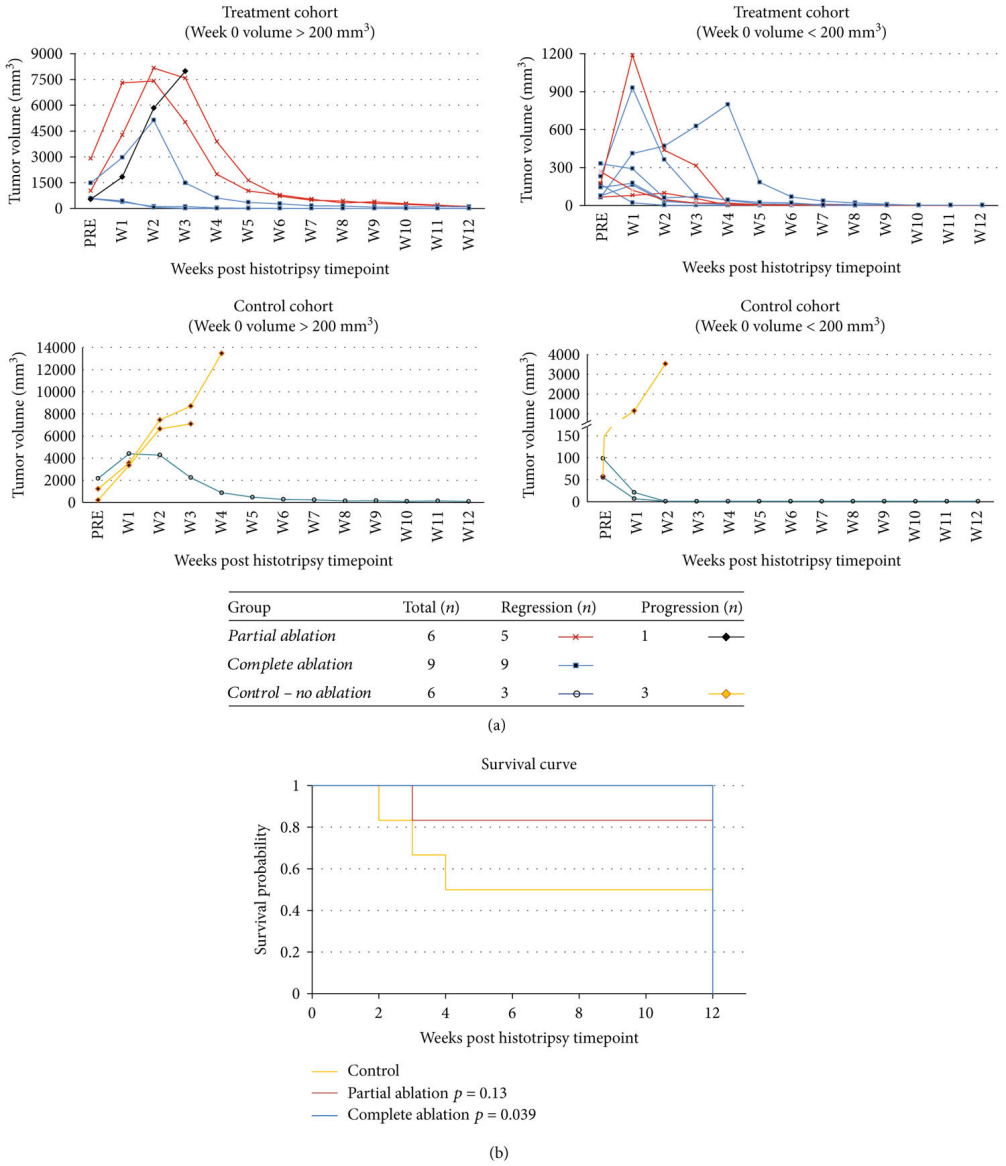
Histotripsy tumor response and survival outcomes. (a) Local tumor regression was observed in 9/9 complete ablation cases and 5/6 partial ablation cases irrespective of large tumor volume (>200mm^3^) or small tumor volume (<200mm^3^) as measured at the pretreatment timepoint (week 0). In contrast, 3/6 control animals demonstrated steady tumor progression while the other 3/6 control animals demonstrated spontaneous regression of their tumors. Individually scaled *y*-axes are used to more clearly illustrate changes in tumor size. (b) Kaplan Meier survival curve comparing survival outcomes in the control, partially ablated, and completely ablated groups. All animals still surviving at 12 weeks were euthanized due to reaching the study endpoint (3 months) in the protocol.

Effective tumor volume reduction with no recurrence was observed in 9/9 completely ablated animals and 5/6 partially ablated animals (Figure 1(a)). The original untreated tumor demonstrated mild hyperintense signal on T2-weighted MRI when compared to the adjacent uninvolved liver parenchyma. Tumor volumes at the pretreatment timepoint ranged from 55.26mm^3^ to 2173.05mm^3^ with 636.49 ± 358.52 (mean ± SEM) in the control group, 67.35mm^3^ to 2900.77mm^3^ with 834.18 ± 436.68 in the partial ablation group, and 64.06mm^3^ to 1476mm^3^ with 401.31 ± 148.28 in the entire ablation group. There were no statistically significant differences in the mean tumor volumes of the three groups at the pretreatment timepoint (*p* = 0.58). Subsequent timepoints are measured from the pretreatment/histotripsy timepoint (week 0).

### 2.3. Survival Outcomes.

In general, rats in the complete ablation group survived significantly longer than the control rats (*p* = 0.039) (Figure 1(b)). Though animals in the partial ablation group had better survival outcomes than the control group, the difference was not statistically significant (*p* = 0.13) (Figure 1(b)). The survival time was 7.5 ± 2.03 (mean ± SEM) weeks in the control group, 12 ± 0 weeks in complete ablation group, and 10.5 ± 1.5 weeks in partial ablation group. The survival time range was 2-12 weeks for the control group, 3-12 weeks for the partial ablation group, and 12 weeks for the complete ablation group after histotripsy timepoint. All survival times are reported at the post histotripsy timepoint, which was two weeks post-tumor inoculation. 9/9 animals in the complete ablation group, 5/6 animals in partial ablation group, and 3/6 animals in the control group were still surviving at 12 weeks, but euthanized due to reaching the study endpoint per our protocol. Thus, the lack of a significant difference in survival time between the partial ablation and control group is in part due to the study endpoint in our protocol. The remaining 1/6 animals in partial ablation group and 3/6 animals in the control group were euthanized at earlier timepoints due to tumor burden greater than 25mm in any one dimension.

### 2.4. Tumor Response to Complete Histotripsy Ablation: MRI Observations.

**Figure 2:**
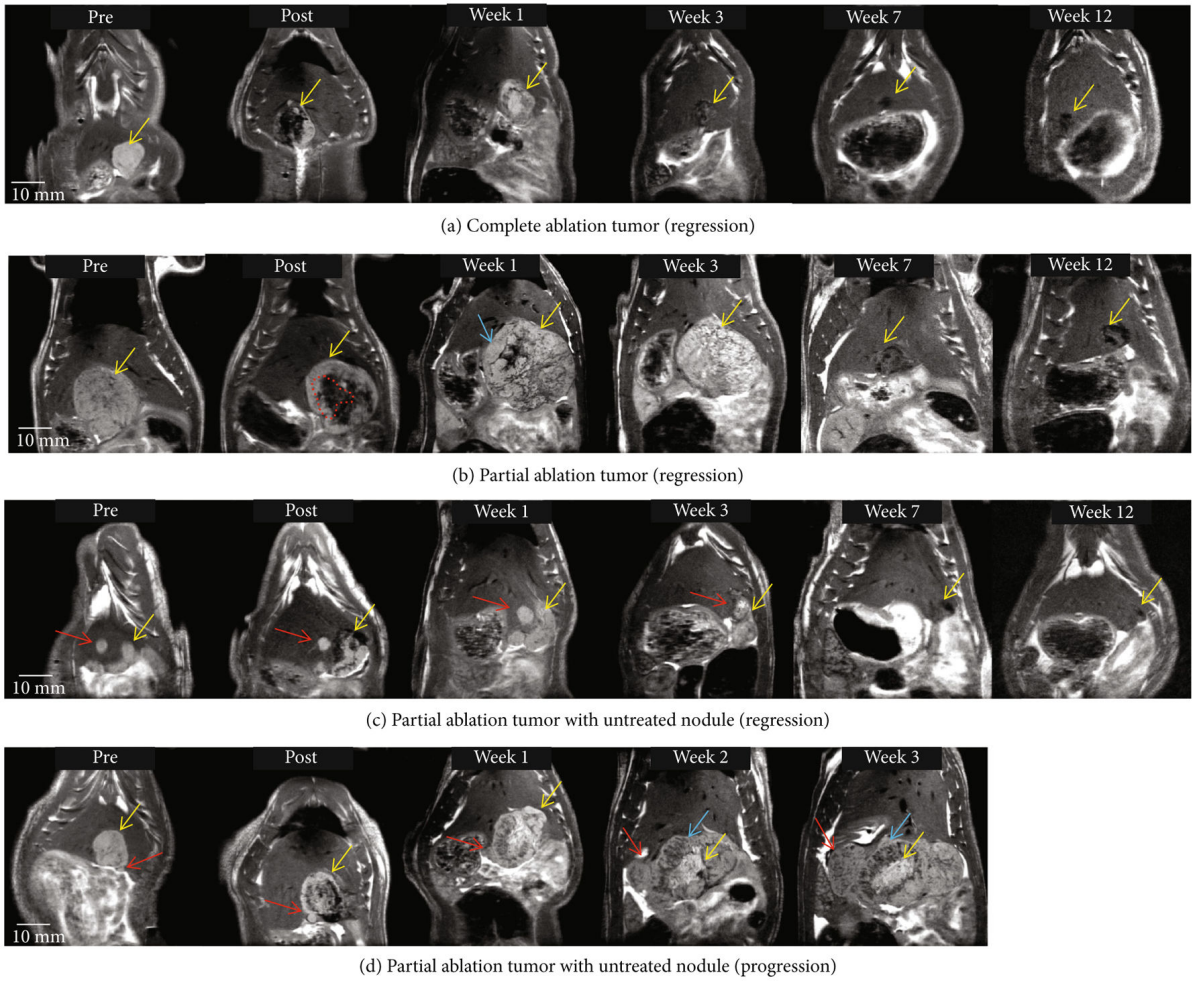
Tumor response to histotripsy on T2-weighted MRI. The size and appearance of tumor (yellow arrow) in response to complete and partial histotripsy ablation are observed at different timepoints. (a) Complete ablation–pre-treatment (mildly hyperintense), posttreatment ablation zone (hypointense), and ablation zone at week 1 (mildly hyperintense), week 3 (mildly hypointense, size regression), week 7 (hypointense, size regression), and week 12 (<5mm hypointense region). (b) Partial ablation–pretreatment (mildly hyperintense), posttreatment ablation zone (hypointense, shown by red dashed lines) and untreated tumor region (mildly hyperintense), and lesion at week 1 (mildly hyperintense) with pseudoprogression characteristics (blue arrow), week 3 (mild hyperintense, size regression), week 7 (hypointense, size regression), and week 12 (~5mm hypointense region). (c) Partial ablation with a separate untreated nodule (red arrow)–ablation zone and untreated nodule at week 1 (mildly hyperintense), week 3 (mildly hyperintense with no size progression), week 7 (hypointense, size regression), and week 12 (<3mm hypointense region). (d) Partial ablation with adjacent untreated nodule (red arrow)–ablation zone at weeks 1-3 shows a central hyperintense zone (yellow arrow) surrounded by heterogeneous hypointense zone (blue arrow). The untreated nodule (red arrow) increases in size over weeks 1-3 and demonstrates mildly T2-hyperintense signal. In animals demonstrating regression, the hypointense region at the original tumor site observed on ~3 month MRI indicates near-complete resolution of the tumor.

Generally, smaller tumors (largest dimension measured < 1 cm) appeared homogeneous, and their appearance became increasingly heterogeneous as the largest dimension measured (>1 cm) (Figures 2 and S1). MRI imaging performed within 6 hours postablation demonstrated T2-hypointense signal at the ablation zone (Figure 2(a)). MR imaging performed ~1 week after histotripsy ablation demonstrated T2 hyperintense signal within the treatment cavity which mimics that of fluid. The T2 hyperintense appearance persisted for up to 3 weeks post histotripsy, gradually becoming less hyperintense and completely transforming to T2-hypointense signal by week 7 as the treatment cavity decreased in size over time indicating tumor regression (Figure 2(a), S1, and S2). At 8-12 weeks post-histotripsy, a 1-5mm hypointense area was observed at the original tumor site, indicating fibrous tissue and calcification remnants, as confirmed by histology. Figure S1 compares the T2W (T2-Weighted) MRI appearance of the post-histotripsy lesion at select timepoints with the appearance of viable pretreatment tumor. Figure S2 shows imaging observations at all weekly timepoints for the complete ablation case demonstrated in Figure 2(a).

### 2.5. Tumor Response to Partial Histotripsy Ablation: MRI Observations.

For the partially treated tumors, the treatment cavity demonstrated a T2 hypointense signal and the residual untreated tumor demonstrated mild T2 hyperintense signal (Figure 2(b)). The hypointense appearance of the ablation zone seen in the early postablation period (<6 hours) allowed for easy delineation between ablated and residual nonablated tumors. MRI also revealed a transient increase in the size of the lesion when compared to the pretreatment tumor size at 1 week post histotripsy (Figure 2(b)), presumably due to inflammation-derived tumor pseudoprogression. The entire lesion began to regress starting at week 3, resulting in near-complete resolution of the tumor homogenate in weeks 4-12 with a ~5mm nontumoral fibrous tissue zone detectable on MRI remaining at the original tumor site at the 3-month timepoint in 5/6 animals (Figure 2(b) and S1).

Of note, two cases demonstrated a separate small nodular tumor adjacent to the targeted tumor, and the separate small tumor nodule was left untreated. On MRI, the untreated tumor maintained the same appearance after treatment as the pretreatment tumor. In the first case (Figure 2(c)), both the residual tumor along the margin of the partially ablated tumor and the adjacent untreated nodule responded and regressed starting at week 3 and eventually resulted in near-complete resolution by week 12. However, in the second case (Figure 2(d)), the untreated nodular tumor continued to increase in size and demonstrated signal characteristics similar to the pretreatment tumor. The treated portion of the tumor demonstrated the expected pseudoprogression at weeks 1-3. The rat was euthanized at week 3 due to increased tumor burden reaching euthanasia limit, and thus, the long-term effect of histotripsy on the untreated nodule could not be investigated. Figures S3 and S4 show imaging observations at all weekly timepoints for the partial ablation cases demonstrated in Figures 2(b) and 2(c).

### 2.6. Tumor Response to No Treatment (Control): MRI Observations.

**Figure 3:**
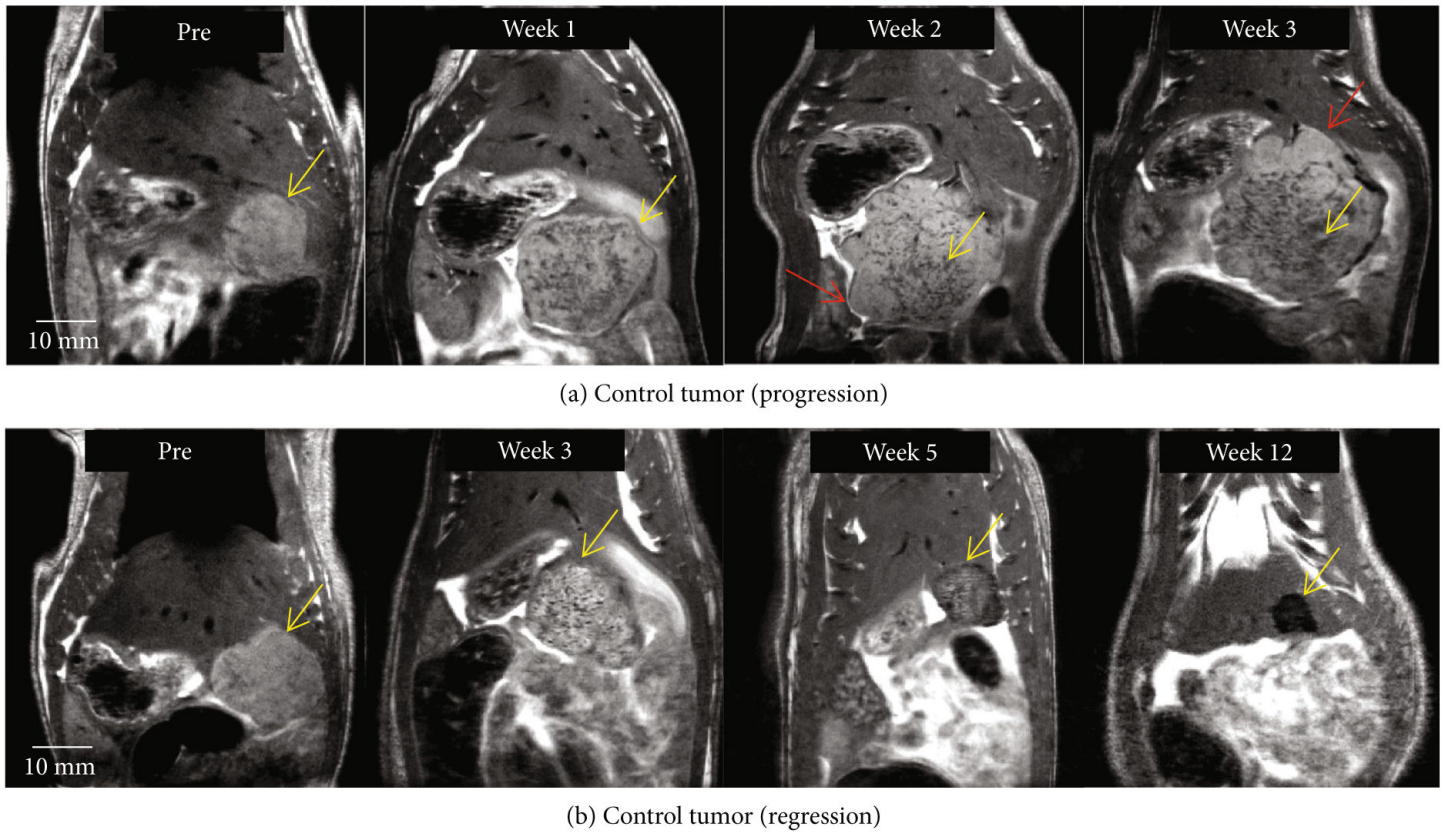
Control tumor evolution on MRI. (a) Control tumor (yellow arrow) demonstrates satellite nodules along the periphery of the primary tumor (red arrow), which eventually contribute to tumor progression. (b) Control tumor (yellow arrow) develops a mottled and heterogeneous appearance, similar to that of pseudoprogression by week 3 and regresses as evidenced by decreasing tumor burden at weeks 5 and 12.

In the control group, tumor burden continued to increase in all animals until two weeks postinoculation. In three rats, control tumors demonstrated satellite nodules along the periphery of the primary tumor which increased in size. The peripheral satellite nodules also continued to develop which contributed to the progressive increase in tumor burden, and the animals were euthanized at by 2-4 weeks (Figure 3(a)). In the other three control animals, tumors developed a diffuse mottled and heterogeneous appearance by week 3 and eventually spontaneously regressed by week 12 (Figure 3(b)). Figure S5 shows imaging observations at all weekly timepoints for the control tumor demonstrated in Figure 3(b).

### 2.7. Effects on Adjacent Tissue.

The original ablation volume in the complete treatment cohort (*n* = 9) included up to a 2mm margin of the normal hepatic parenchyma, to ensure complete ablation of potentially microscopic disease. Posttreatment imaging in all subjects demonstrated “pseudo-progression” of the ablated tumor, in which the entire lesion increased in size, likely a combination of immediate posttreatment edema and the acellular homogenate which was created by histotripsy ablation. This was resolved within 2-3 weeks posttreatment in all subjects demonstrating regression. There was no evidence of immediate post-histotripsy tissue damage along the treatment path outside the treatment zone, as seen on MR imaging (i.e., no T2 signal changes). Additionally, there were no gross changes seen on the skin surface in any of the animals treated by histotripsy. Apart from the residual fibrous scar at the treatment zone, there was no imaging or histopathologic evidence of parenchymal atrophy surrounding the ablation zone. Finally, there was no evidence of intrahepatic biliary duct dilatation upstream from the ablation zone, or vascular thrombosis (no signal detected within the vessels on T2 imaging), although lack of contrast did limit evaluation.

### 2.8. Histological Analysis.

**Figure 4:**
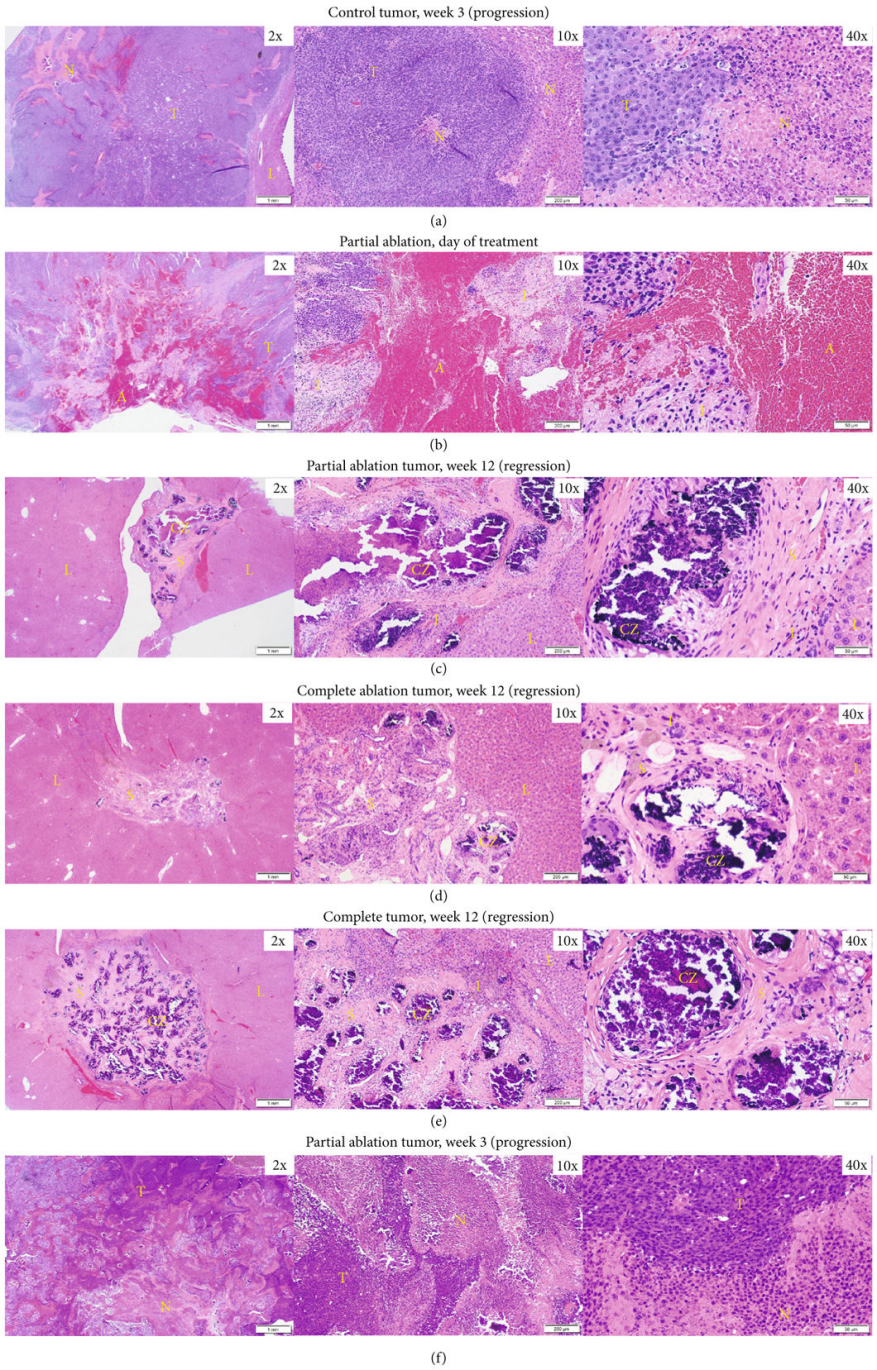
Representative histology images. (a) Control HCC at 3 weeks demonstrating progression shows viable tumor (T) with prominent region of necrosis (N). (b) Partial ablation on day 0, the ablation zone (A) is comprised mainly of extravasated red blood cells and fibrin, and is surrounded by regions of inflammatory cells (I) and regions of viable tumor (T). (c) Partial ablation at 12 weeks demonstrating tumor bed completely replaced by scar tissue (S) and scattered areas of dystrophic calcification (CZ) within the ablated region with a thin rim of inflammatory cells (I) separating the ablated zone from normal liver (L) and no evidence of viable tumor. (d) Complete ablation at 3 months demonstrating tumor regression and replacement by scar tissue (S) with scattered dystrophic calcification (CZ) and a thin rim of inflammatory cells (I). No evidence of viable residual tumor in the surrounding normal liver (L). (e) Control tumor at 3 months demonstrating complete tumor regression, scattered inflammatory cells (I), surrounding scar tissue (S), and dystrophic calcification (CZ) at the original tumor site. No evidence of viable residual tumor in the surrounding normal liver (L). (f) Partial ablation at 3 weeks demonstrating tumor progression. Viable tumor (T) is observed with some regions of necrosis (N). Timepoints are measured from the histotripsy treatment time as week 0.

In untreated tumors in the control group that did not regress (*n* = 3/6), prominent regions of necrosis were observed at week 3 (euthanasia timepoint) (Figure 4(a)). In the acute study group (*n* = 2), the treated region was composed of extravasated blood cells, fibrin, and inflammatory cells on day 0 (Figure 4(b)). In the partial ablation survival group, in animals demonstrating regression (*n* = 5/6), histological analysis revealed scar tissue (<5mm) with scattered dystrophic calcification within the ablated region and a submillimeter rim of inflammatory cells separating the ablated zone from the normal liver at 3 months (euthanasia timepoint) (Figure 4(c)). No residual viable tumor cells were observed. Similar to the partial ablation, in the completely ablated group (*n* = 9/9), the ablation zone also demonstrated scar tissue (<5mm) with scattered dystrophic calcification and inflammatory cells and no evidence of viable tumor at 3 months (euthanasia timepoint) (Figure 4(d)). These histological observations correlate with the MRI observation of <5mm T2-hypointense tissue at the original treatment site, indicating complete regression of these tumors to form residual scar tissue. In control rats demonstrating regression (*n* = 3/6), no residual tumor cells were observed, with similar findings of scattered inflammatory cells surrounding scar tissues and calcification at the original tumor site (Figure 4(e)). In the partial ablation case where the tumor did not regress (*n* = 1/6), the tumor showed necrosis regions at week 3 (Figure 4(f)).

### 2.9. Effects on Metastases.

No metastases were detected within the liver by gross or microscopic evaluations in any of the ablation groups. However, the N1-S1 tumor model does not naturally develop metastasis, and our results support that histotripsy did not yield unexpected intrahepatic metastasis.

## 3. Discussion

This study evaluated the local tumor progression of orthotopic HCC in response to a single histotripsy treatment in an immune-competent, N1-S1 rodent liver tumor model. The N1-S1 rodent tumor model is one of the most common rodent orthotopic HCC models used for image-guided interventional oncology research [30, 31]. Histotripsy has been studied extensively to treat normal liver tissue in human-scale porcine models [23, 27, 28]. To our knowledge, this is the first study investigating histotripsy ablation of orthotopic, liver tumors in immunocompetent rats with a long-term (3 months) follow-up.

Histotripsy ablation responses were evaluated with MRI and histology. Our results demonstrate that histotripsy resulted in effective local tumor burden reduction with no local recurrence in 14/15 treated rats which demonstrated complete regression of the original ablated tumor. The ablated volume was nearly completely resorbed (with only a small focus of residual fibrous scar remaining at original site) in 5/6 rats which underwent partial tumor ablation and 9/9 rats which underwent entire tumor ablation. In these animals, the appearance of the ablated volume had transformed completely to T2-hypointense (indicative of nontumoral scar tissue) by week 7 and there were no further observable changes in its appearance until the final imaging timepoint at week 12 when it was confirmed by histology that there was no viable tumor. No histotripsy-treated animals demonstrated intrahepatic metastases.

MRI has been utilized to characterize tumor response and to quantify tumor necrosis in pre-clinical animal and human tumor models as well as for the clinical assessment of locoregional therapies [16, 32]. In this study, post-histotripsy MRI results of partially ablated tumors demonstrated clear delineation between treated tumor and residual viable tumor, as seen by differences in their T2 signal (Figures 2(b), 2(c), and 2(d)). Thus, histotripsy therapeutic response can be assessed by MRI immediately postablation, as the signal characteristics of the pretreatment tumor and the ablated zone are distinct. These characteristics are essential for early post-HCC treatment response evaluation, as the immediate detection of the residual tumor can allow for follow-up retreatment to ensure adequate ablation. This is a limitation of current practices with some forms of locoregional therapy for HCC (particularly arterial and radiation-based locoregional therapy (TACE, TARE, and SBRT)), in which treatment effectiveness can only be adequately assessed 4-12 weeks posttreatment [33].

MR imaging also demonstrated tumor pseudoprogression (transient increase in the size of the targeted tumor) following histotripsy, seen as an early enlargement of the treatment cavity before subsequently regressing, as evidenced by decreasing the size of the treatment zone and eventual conversion to a small T2 hypointense fibrous nodule. Although the treatment cavity size transiently increased in the early posttreatment time period (up to 3 weeks), the MR imaging appearance of the treatment zone remained distinct from the residual viable tumor, as seen in the partial ablation group. The imaging findings of pseudoprogression are usually seen in tumors treated with systemic immunotherapy (e.g., melanoma, renal cell carcinoma, and squamous cell lung carcinoma), although pseudoprogression has also been reported after locoregional ablation therapies as well as immunological therapies for HCC [34-36]. Previous studies have indicated that an immunologic response to treatment may induce the infiltration of immune mediators and subsequent inflammation within the tumor, which results in the appearance of tumor pseudoprogression [3, 35]. Though histotripsy relies on mechanical cavitation to destroy tumor cells and is not a pure immunologic treatment, the findings of immune cell infiltration surrounding the treatment cavity as seen on histology posttreatment, as well as the increased tumor size 1-3 weeks post-histotripsy on MRI evaluation, suggest pseudoprogression may be the initial response to histotripsy, followed by the antitumor response resulting in regression of the viable untreated tumor. These results are consistent with the regression of untreated tumors in 5/6 of the partially treated cases. Our recent study has demonstrated that histotripsy ablation of subcutaneous murine B16GP33 melanoma tumors in immunocompetent C57BL/6 murine hosts releases tumor antigens with preserved immunogenicity resulting in a potent local and systemic immunostimulatory response, which reduced distant untreated tumors [37]. In the poorly immunogenic Hepa 1-6 subcutaneous liver tumor model with immunocompetent C57BL/6 murine hosts, histotripsy by itself and in combination with checkpoint inhibition immunotherapy stimulated abscopal immune effects at distant tumor sites [37]. In comparison, in a separate study on immunocompromised mice bearing Hep3B human cell line-derived subcutaneous flank tumors, histotripsy tumor ablation resulted in near-complete resorption of the ablation zone. However, due to a lack of sufficient margin along the skin-ablation zone interface, residual viable tumor cells were observed resulting in eventual tumor recurrence in the immunocompromised mice [29]. Further studies are planned to explore the specific immunomodulation mechanisms in response to histotripsy in orthotopic liver tumor models and directly compare the response in immunocompromised vs immunocompetent hosts.

One confounding result in this study is that 3/6 control rats demonstrated spontaneous regression of their tumors despite lack of treatment, although the other 3 control animals demonstrated steady tumor progression and had to be euthanized due to increased tumor burden. Other studies have also reported that N1-S1 tumors may automatically regress after 5-6 weeks [30]. The presence of an intact immune system may have contributed to the regression of the tumor in otherwise healthy subjects. Further studies, with larger numbers of controls, using more aggressive and highly-metastatic HCC tumor models, are needed in order to understand the effects of histotripsy on intrahepatic and distant metastases and the eventual impact on disease-free survival outcomes.

This study revealed no irreversible damage to structures both adjacent to the ablation zone and along the ablation path. In instances of complete ablation where we attempted to define a margin outside the tumor region, posttreatment and weekly follow-up MRI revealed edema in the liver surrounding the treatment region, manifested as a T2-hypointense signal. Despite deliberate ablation of healthy liver tissue to ensure an adequate ablation margin, post-histotripsy imaging revealed no atrophy within the parenchyma adjacent to and upstream from the ablation zone, confirmed with gross pathology and histology. Furthermore, there were no abnormal signals or imaging findings seen on MRI along the cavitation pathway from the surface of the skin to the tumor within the liver, and even deep to the tumor. There was no intrahepatic biliary ductal dilatation within the parenchyma upstream from the ablation zone and no evidence of vascular injury including bleeding or venous thrombosis (although lack of intravenous contrast may have limited evaluation). These findings are supported by previous large animal studies that have indicated no damage to the veins near the histotripsy ablation zone or other off-target tissues while treating the liver [28].

There are a few other limitations of this study, primarily related to the choice of tumor model. First, N1-S1 cells implanted orthotopically do not fully mimic tumors that develop spontaneously in a cirrhotic liver. Since the N1-S1 cell line is of nonhuman origin, it does not fully represent human HCC, thus limiting the ability to translate this data to human HCC. Interhost tumor growth variability was also observed at the pretreatment timepoint as tumorigenesis is a complex process involving interactions with the immune system. As injection of cyclophosphamide was performed 24 h prior to tumor inoculation to support tumor formation by initiating temporary immune suppression [38], there may have been differences in the immunosuppression action across hosts as evidenced by the observed tumor volume heterogeneity at two weeks postinoculation. Despite this limitation, an immunocompetent rat is necessary in order to study the role of the immune system in the effects of histotripsy on the local tumor. And using a human HCC cell line in an immunocompromised rodent model would limit the investigation of an immune-mediated response to histotripsy. Another limitation of this study is the use of basic MR sequences without intravenous contrast agents or diffusion-weighted imaging (DWI) sequences. Further studies using additional MR sequences to evaluate pre- and histotripsy-treated tumors at varying time intervals postablation are necessary to better understand imaging features post-histotripsy. DWI sequences can help evaluate posttreatment MR signal transformations, allowing clearer differentiation between local tumor progression versus post treatment tissue changes, such as early pseudoprogression [39]. Contrast enhancement on MRI with liver-specific contrast agents may further enhance detection of ablation-induced effects such as vascular obstruction, edema, and tumor necrosis [40].

Overall, this study demonstrated the potential of histotripsy for noninvasive tumor ablation in an immunocompetent rodent HCC model with a high safety profile and low risk of local tumor progression. The study also characterizes the MR imaging observations demonstrating treatment response for up to 3 months following treatment. Local tumor shrinkage was observed in 14/15 (93.3%) treatment rats with no recurrence at 3 months. Additionally, preliminary data showed complete tumor regression despite only partial tumor treatment. The lack of injury to adjacent organs or tissue along the treatment pathway demonstrated a high safety profile for histotripsy. Furthermore, this study suggests a possible immune-mediated response stimulated by histotripsy, resulting in local tumor cell destruction. Research is ongoing to further evaluate the effects of primary tumor histotripsy ablation on distant metastatic tumors. Future studies will continue to investigate the safety, efficacy, and biological effects of histotripsy for potential translation to the clinic.

## 4. Materials and Methods

### 4.1. Experimental and Technical Design.

Orthotopic N1-S1 hepatic tumors were developed in immunocompetent Sprague-Dawley rodent hosts. Liver tumors were targeted for histotripsy ablation either partially (50 - 75% tumor volume) or completely (100% tumor volume), and animals from both groups were monitored for up to 3 months by MRI for local tumor response and examined by histological evaluation at study endpoint. Additionally, MRI and pathology were used to evaluate for distant intrahepatic metastasis following histotripsy ablation. Primary tumor growth characteristics, tumor response to histotripsy, MR imaging appearance, and survival outcomes were assessed.

**Figure 5:**
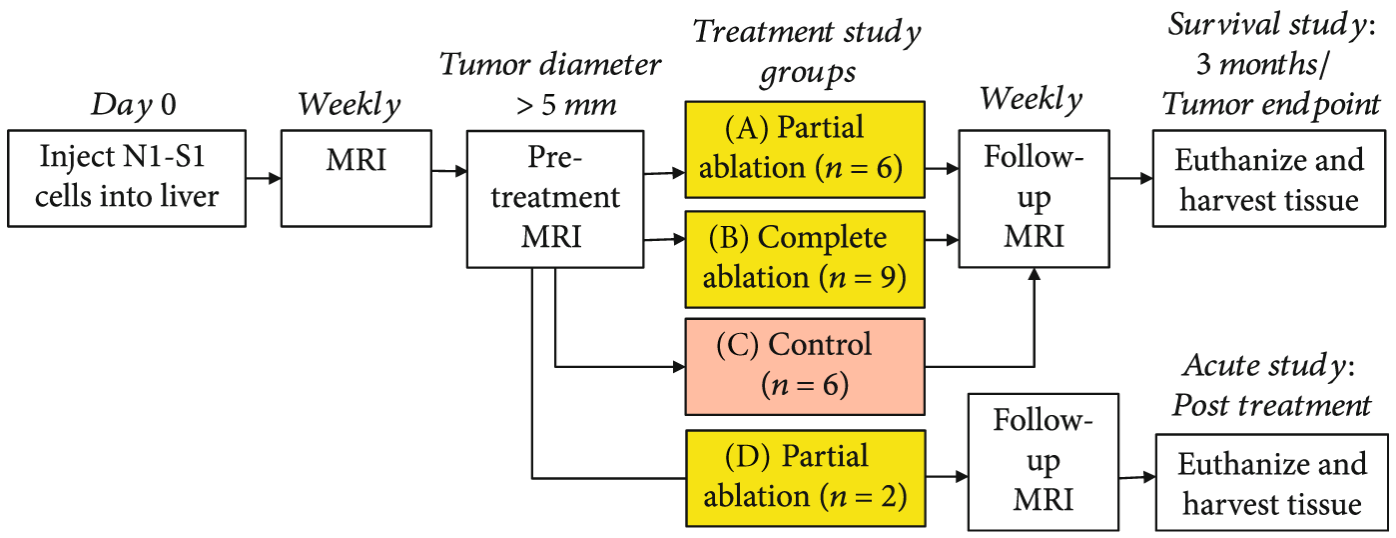
Study protocol. Histotripsy ablation (either partial or entire tumor) was performed once the tumor measured a minimum of 5mm in the largest diameter. The survival groups A, B, and C were survived and monitored weekly using MRI for up to 3 months post treatment or until the maximum tumor diameter exceeded 25 mm. The acute rats (group D) were euthanized immediately after ablation. For the partial ablation groups A and D, 50-75% of the tumor volume was ablated by histotripsy. In the entire tumor ablation group B, the entire tumor with additional margin was targeted for ablation. Animals in control group C received no treatment.

The study protocol is shown in Figure 5. N1-S1 tumor cells were injected in the inferior liver lobe of 30 Sprague Dawley rats weighing 200-250 g. In the survival study, the rats were categorized into three groups based on the tumor volume targeted for ablation: (A) partially targeted (*n* = 6), (B) entirely targeted (*n* = 9), and (C) control (*n* = 6). To demonstrate the acute response, a fourth group of rats, D (*n* = 2), was euthanized immediately after histotripsy. MRI and pathology were performed on the acute rats. Five rats did not develop primary tumors and were thus excluded from the study. Two rats developed tumors < 5mm in diameter and were also excluded from the study. Histotripsy ablation (either partial or complete) was performed once the tumor measured a minimum of 5mm in its largest diameter. Rats in the survival study were monitored weekly post ablation using MRI for up to 3 months or euthanized when the maximum tumor diameter exceeded 25mm. For the partial ablation groups, approximately 50-75% of the tumor volume was targeted for ablation by histotripsy. In the entire tumor ablation group, the entire tumor was targeted for ablation, with an additional small margin (<2mm) of hepatic parenchyma beyond the tumor boundary included in the target region. The control rats received no treatment.

### 4.2. Animals and Study Approval.

This study was approved by the Institutional Animal Care and Use Committee at the University of Michigan (UM-IACUC, Protocol Number: PRO00007764). The UM-IACUC has specifically approved the internal orthotopic tumors to grow to a 25mm maximum diameter size, provided that none of the end-stage illness criteria are reached and the animals are monitored weekly using MR imaging. End stage illness and humane endpoints guidelines were determined as follows: animals exhibiting signs of dehydration or emaciation or cachexia, impaired mobility, systemic infection, or abdominal or thoracic bleeding during, or immediately after histotripsy treatment were euthanized. The primary method of euthanasia was exposure to CO_2_ in a compressed gas form followed by a secondary method of euthanasia, which consists of inducing bilateral pneumothoraces to ensure death, in accordance with UM-IACUC guidelines.

Thirty Sprague Dawley rats weighing 200-250 g were purchased from Taconic (Hudson, New York) and housed and maintained in specific pathogen-free (SPF) conditions in University of Michigan ULAM (Unit for Laboratory Animal Medicine) housing facility. ULAM technicians provided daily animal husbandry care. Animals were housed in ventilated cages with clean nesting material. Animals were housed in biocontainment housing upon the injection of cyclophosphamide and were moved back to regular SPF housing after 3 days, in accordance with the UM-IACUC protocol. Animals were provided with rodent standard lab diet and autoclaved water bottles.

### 4.3. Cell Preparation.

N1-S1 (ATCC^®^ CRL-1604™) cells were obtained directly from the ATCC cell line repository and were cultivated in Iscove’s Modified Dulbecco’s Medium (IMDM) containing 4mM L-glutamine, 4500mg/L glucose, and 1500mg/L sodium bicarbonate, supplemented with 10% FBS, 1% antimycotic-antibiotic and 1mL Gentamicin. The cells were maintained at 37°C in a 5% CO_2_/95% humidified air atmosphere. The N1-S1 (ATCC^®^ CRL-1604™) cell line has been originally established from a hepatoma induced by feeding 4-dimethylaminoazobenzene to a male rat.

### 4.4. Orthotopic Tumor Inoculation Procedure.

All orthotopic tumor inoculation surgeries were performed in accordance with UM-IACUC approved protocol. Twenty-four hours prior to tumor inoculation, rats were administered 100 mg/kg cyclophosphamide via intraperitoneal injection. Prior to and during surgery, the rats were induced and maintained on general anesthesia by inhalation of isoflurane gas (1.5-2.0%) admixed with 1 L/min of oxygen (SurgiVet V704001, Smiths Medical, Waukesha, Wisconsin, USA). A small animal monitoring system was used to monitor the rectal temperature, heart rate, and oxygenation (SpO_2_) levels throughout the surgery. Core body temperature was maintained between 35 and 37°C with the use of a heating pad and an overhead heating lamp. Sterile field was maintained during surgery, and autoclaved instruments were used. The rats were injected with Carprofen (Rimadyl, Pfizer, NY, USA) analgesic (5 mg/kg) subcutaneously before surgery and once every 24 hours for 2 days after surgery for analgesia. The hair covering the chest and abdomen was removed with an electric clipper. The surgical area was sterilized by scrubbing with chlorhexidine spray, a wet 4 × 4 gauze, and iodine. To expose the liver, an open laparotomy was performed by making a midline incision through the skin and the abdominal wall. Using forceps and spreader, the left liver lobe was isolated. Two to four million N1-S1 cells suspended in 0.1mL Matrigel + PBS (phosphate-buffered saline) were injected into the inferior portion of the left lobe, and pressure was applied on the injection site using sterile cotton tip applicators to prevent leakage. The muscle layer was closed using absorbable sutures, and the skin layer was closed using staples. Animals were transferred to a recovery cage and were monitored until ambulatory; thereafter, they were returned to their housing cage. Approximately one week postsurgery, the skin staples were removed. All rats were monitored weekly and tumor diameter measurements were quantified using MRI. Once tumor diameters reached 5mm in the largest dimension, the rats in the treatment groups were treated with histotripsy. Rats in the control group did not receive histotripsy or other treatment.

### 4.5. Animal Preparation for Histotripsy.

Prior to and during histotripsy, the rats were induced and maintained on general anesthesia by inhalation of isoflurane gas (1.5-2.0%) in 1 L/min of oxygen (SurgiVet V704001, Smiths Medical, Waukesha, Wisconsin, USA). The hair covering the chest and abdominal region was removed with an electric clipper. Core body temperature was recorded using a rectal probe and was maintained between 35 and 37°C with an overhead heating lamp. Rats were injected with Carprofen (Rimadyl, Pfizer, NY, USA) analgesic (5 mg/kg) subcutaneously before delivery of histotripsy treatment and once every 24 hours for 2 days after treatment. Immediately after histotripsy, the rats were transferred to a recovery chamber fitted with an overhead heating lamp, and were placed on a heated pad. Once the rats recovered from anesthesia and were mobile, they were transferred back to their housing cages. The rats were monitored weekly for the duration of the study. Tumor dimensions and the treatment response were monitored using MR imaging. Animals were survived up to 3 months or until the tumors reached a diameter of 25mm in maximum diameter (tumor endpoint).

### 4.6. Rodent Histotripsy Setup.

**Figure 6:**
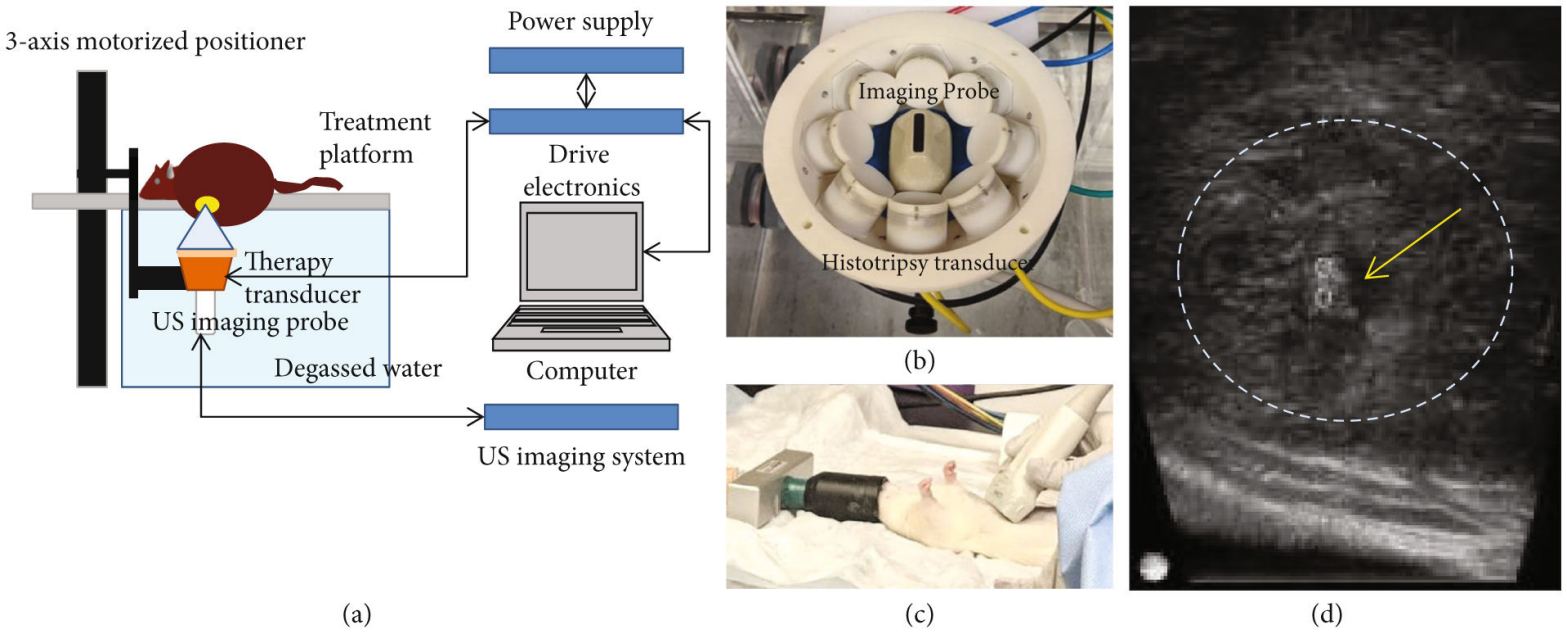
Histotripsy setup and procedure. (a) Histotripsy therapy transducer, driven by custom-built driver electronics, is interfaced with a computer which delivers histotripsy pulses to generate focal cavitation. Single focus is scanned to cover desired target volume by using motorized positioner to control movement of the therapy transducer and the imaging probe. (b) The 1MHz histotripsy therapy transducer constructed as an 8-element ring configuration with the coaxially aligned imaging probe in the center. (c) Ultrasound imaging of the rat liver and tumor was performed prior to placing the rat on the treatment platform to determine tumor location. The tumor location is marked before the rat is placed on the treatment platform, which contains an aperture to allow the tumor region intended for treatment to be immersed in water (ultrasound coupling medium). (d) Generation of hyperechoic cavitation cloud (yellow arrow and two small circles mark the axial extent of the cavitation) in liver tumor (dashed lines).

Our lab has designed and built a customized 8 element 1MHz focused ultrasound therapy transducer specifically for rodent histotripsy therapy [20]. The transducer (*f* number = 0.6) was designed as a ring configuration scaffold consisting of 8 individually focused lead zirconate titanate elements (20mm diameter) operating at 1MHz with a focal distance of 32.5mm using stereolithographic rapid prototyping techniques as discussed by Kim [41]. The histotripsy transducer was driven by a custom-built high-voltage pulser controlled by a field-programmable gate array (FPGA) development board (DE0-Nano Terasic Technology, HsinChu, Taiwan) (Figure 6(a)). The electronical driving signal to the histotripsy therapy transducer was a 1-cycle pulse. The acoustic waveform generated by the histotripsy transducer contained a single dominant negative pressure phase. Histotripsy bubble cloud is produced when the maximum negative amplitude exceeds a distinct pressure threshold (intrinsic threshold) inside the tissue (~26MPa for liver) [18, 42]. This intrinsic cavitation threshold depends primarily on the negative pressure phase of the pulse and is not affected by the positive pressure phases of the histotripsy pulses [19]. In free field, the transducer generated an estimated focal peak negative pressure > 30MPa based on pressure measurements from fiber optic hydrophone [20]. A 20MHz B-mode ultrasound imaging probe (L40-8/12, Ultrasonix, Vancouver, Canada) was coaligned with the center of the therapy transducer to allow visualization of the focal ablation volume in real time (Figure 6(b)). The ultrasound therapy transducer and the imaging probe were mounted to a motorized 3-axis positioning system (stepper motor: 17MDSI102S, Anaheim Automation, Anaheim, CA, USA and linear motion system: MS33-100218, Thomson Linear Motor System, Radford, VA, USA) to mechanically scan the therapy focus across a 3D target ablation volume (Figure 6(a)). The manufacturer precision for the linear motion system is specified as 5 *μ*m, and the stepper motor step size resolution is listed as 0.225°. Water was used as the ultrasound coupling medium by immersing the transducer and imaging probe in a tank of degassed water maintained at 35–37°C by means of a coil heater. The rat was placed on a custom-built animal platform just over the water tank.

### 4.7. Histotripsy Ablation Procedure.

To identify the therapy focus on ultrasound imaging, test histotripsy pulses were delivered to the water tank by the therapy transducer to generate a “bubble cloud” (elliptical shape of approximately 1 × 3mm in water) which appeared as a hyperechoic cavitation zone on ultrasound imaging. The position of the resultant small focal hyperechoic cavitation zone was then marked as the therapy focal position on the ultrasound image.

After sedation, the animal was placed supine and dedicated real-time ultrasound grayscale imaging was performed over the liver to identify the location and size of the tumor (Figure 6(c)). The approximate location was marked with an “X” with a washable marker. Thereafter, the animal was placed prone on the treatment table with the abdominal region submerged in the degassed water. The therapy transducer was then moved by the motorized positioning system to align the therapy transducer focus with the center of the tumor on the ultrasound image. All tumors were measured by real-time ultrasound imaging, and correlation was made with the most recent MRI to ensure accurate size measurement and treatment of adequate tumor volumes. The boundaries of the intended treatment region in 3 dimensions (axial, lateral and elevational) were manually determined on ultrasound imaging, and an ellipsoid “volume” was generated from these user-defined boundary values using a custom MATLAB script. This treatment ellipsoid encompassed the 3D tumor volume to be targeted for ablation and consisted of a grid of uniformly spaced therapy focal zones (~0.5–0.75mm (lateral, elevational directions) and ~0.75–0.9mm (axial direction)) between adjacent focal volumes (Figure S6). This target volume, as defined on ultrasound imaging, covered approximately 50-75% of the original tumor volume for partial ablation or the complete tumor with additional margin (up to 2mm) for entire ablation. The transducer focus was mechanically scanned (using the motorized positioning system to move the therapy transducer and the imaging probe) to follow the MATLAB-generated grid of focal locations within the treatment ellipsoid. At each focal location, 50 histotripsy pulses at 100 Hz PRF (*p* – ≥30 MPa) were delivered, which equate to 0.5 seconds of ablation time. After ablation, the targeted region appeared hypoechoic on ultrasound imaging, enabling real-time feedback. During ablation, histotripsy-induced cavitation was hyperechoic in appearance compared to the surrounding tissue on ultrasound imaging (Figure 6(d)), which was used to monitor the ablation. Selection of histotripsy parameters (Table 1) was based on results of a previous in vivo and in vitro work done in our lab [43].

### 4.8. MRI for Evaluation of Histotripsy Ablation.

MRI data was obtained within 1 day prior to histotripsy and within 1 day post-histotripsy, followed by weekly imaging to assess the effects of tumor ablation and characterize MR signal variations in tumor appearance. Imaging was performed on a 7.0 T MR small animal scanner using a Direct Drive console (Agilent Technologies, Santa Clara, CA, USA) with a 60mm innerdiameter transmit-receive radiofrequency (RF) volume coil (Morris Instruments, Ontario, Canada) and 70mm innerdiameter transmit-receive RF volume coil (Rapid 70, RAPID MR International, Ohio, U.S.A.). Prior to and during imaging, the rats were induced and maintained on general anesthesia by inhalation of isoflurane gas (1.5-2.0%) in 1 L/min of oxygen (SurgiVet V704001, Smiths Medical, Waukesha, Wisconsin, USA). Respiration was monitored, and animal temperature was maintained at 37 ± 0.5°C using a rectal temperature probe and a custom-built proportional-integral-derivative (PID) controller (LabVIEW, National Instruments, Austin TX) interfaced with a commercially available small animal system (SA Instruments, Stony Brook, NY). Initial pilot scans were performed to confirm positioning. Thereafter, a 2D T2-weighted fast spin-echo (FSE) with respiratory gating was used to visualize the tumor in the coronal plane (Table 2). Intravenous contrast agents were not administered in this study.

The overall signal characteristics of the pretreatment and posttreatment tumor using T2-weighted FSE were recorded and compared to each other to identify potential changes which would differentiate nonviable tumor from viable tumor. MR images were analyzed (Analyze 11.0, AnalyzeDirect, Overland Park, KS) by a radiologist (MML, 13 years of clinical image interpretation experience) in consultation with an imaging scientist (JMG, 20 years of experience in high-field MRI and preclinical small animal models). Tumor volume and postablation homogenate volumes were quantified by summing area measurements made from each slice of the 2D T2-weighted FSE acquisition. Tumor diameter measurements were also made on the T2-weighted sequence in orthogonal anterior-posterior (coronal) and superior-inferior (axial) dimensions. Signal characteristics of the pretreatment tumor and the post-treatment ablation zone were also recorded. Surrounding structures such as the musculature and body wall were evaluated for signs of injury. MR evaluation of the surrounding hepatic parenchyma, overlying histotripsy pathway from the skin to the targeted lesion, and distant sites was also performed to evaluate for local injury or distant metastasis.

### 4.9. Histological Analysis.

Post-euthanasia, treated tumor and liver tissue samples were harvested and fixed in 10% buffered formalin for histopathological analysis. Fixed tissue samples were submitted to ULAM-IVAC (Unit for Laboratory Animal Medicine-In Vivo Animal Core) for paraffin embedding as well as staining. Samples were sectioned in 5 micron slices and stained with hematoxylin and eosin (H&E). Slides were examined by a board-certified liver pathologist (JS) for histopathologic analysis, under a light microscope (Olympus BX43) which was used to capture images.

### 4.10. Statistical Analysis.

Experimental data were analyzed using Microsoft Excel (2013). Pretreatment timepoint tumor volumes in each survival group (control, partial ablation, and complete ablation) were compared with the single-factor ANOVA test, with significance defined as *p* < 0.05. Survival analysis was performed using the Kaplan-Meier method. Survival time data of control and treated rats in each group were compared with the two-tailed Student’s *t*-test, with significance defined as *p* < 0.05.

## Supplementary Material

Supplementary material

## Figures and Tables

**Table 1:** Histotripsy ablation parameters.

Parameters	Value
Histotripsy pulse length	1-2 cycles
PRF (pulse repetition frequency)	100 Hz
Estimated peak negative pressure at focus	>30 MPa
Number of treatment locations in target volume	2000-3000
Number of histotripsy pulses delivered to each location	50
Treatment location spacing within target volume	0.5-0.7mm (lateral, elevational), 0.75-0.9mm (axial)
Treatment time	10-15 minutes based on the targeted volume

**Table 2:** MR acquisition parameters.

Parameters	Value
TR (ms) *	2000
TE_eff_ (ms)	10
ESP (ms)	10
ETL	8
FOV (mm^2^)	60 × 60
Kzero	2
Data matrix (zero-filled)	256 × 256 (512 × 512)
Voxel size (*μ*m^3^)	117 × 117 × 1000
Slice thickness (mm)	1
Number of image slices acquired per scan*	15, 20, 21, or 28
Slice orientation	Coronal
Scan time (minutes)	~8

TR = Time to Repetition; TE_eff_ = Effective Echo Time; ESP = echo spacing; ETL = echo train length; FOV = field of view.

* In majority of scans (>80%), 21 image slices were acquired. In the only scan acquiring 28 image slices, TR was set to 3000 ms.
