# Supplementary material for: Effects of Histotripsy on Local Tumor Progression in an in vivo Orthotopic Rodent Liver Tumor Model
Source: BME Front. Author manuscript; Available in PMC 2021 Jul 28. (PMC8318009; doi:10.34133/2020/9830304)
Supplement: Supplementary file 1 [file NIHMS1666891-supplement-Supplementary_material.pdf]

SUPPLEMENTARY MATERIALS

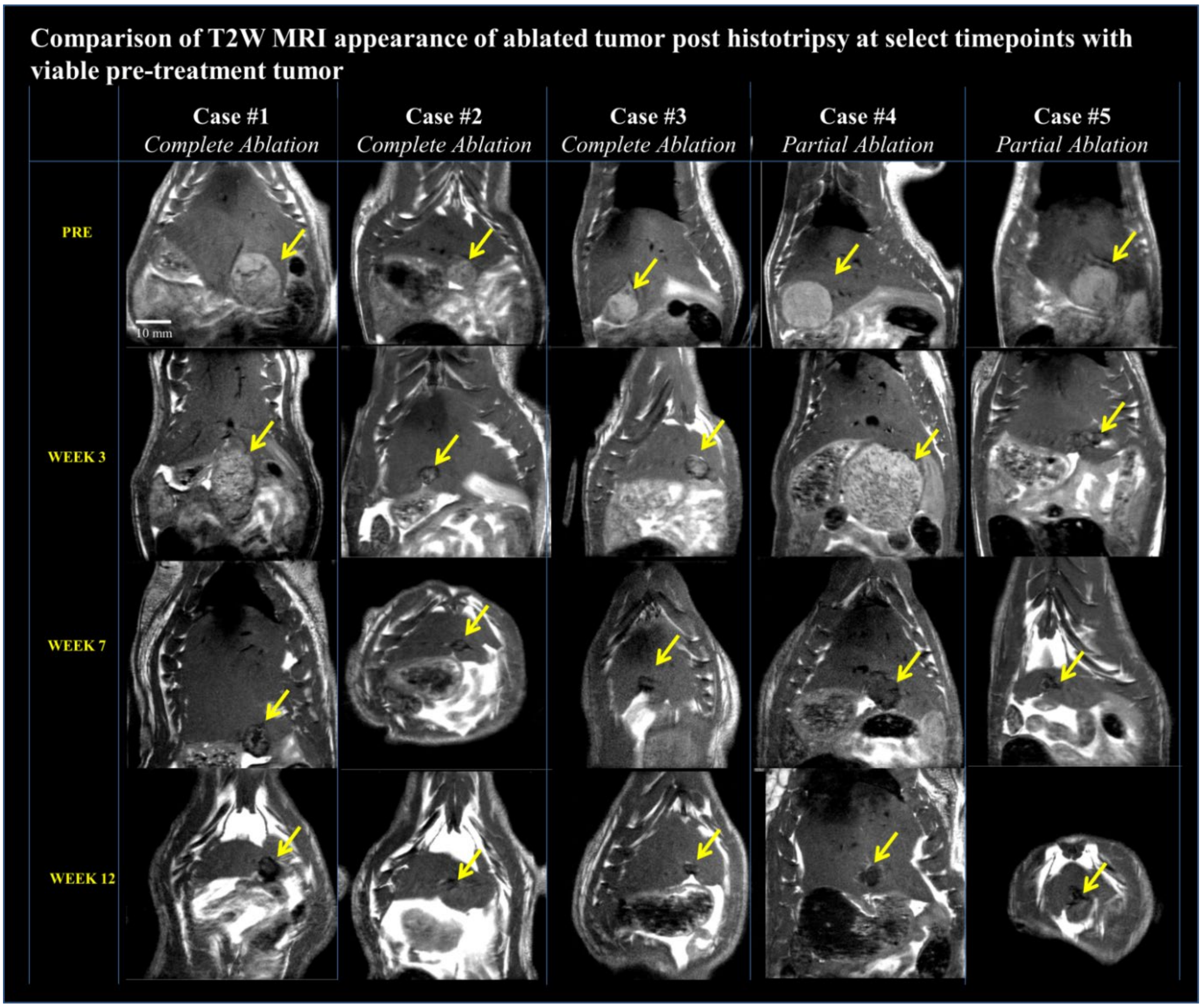

Figure S1. **T2W MRI appearance of histotripsy treated tumor at select timepoints compared to pre-treatment viable tumor for complete and partial ablation cases.** At week 3, the lesion begins to regress, resulting in T2 hypointense signal at the ablation site by week 7. The tumor continues to regress until week 12, but there is no further observable change in signal compared to week 7.

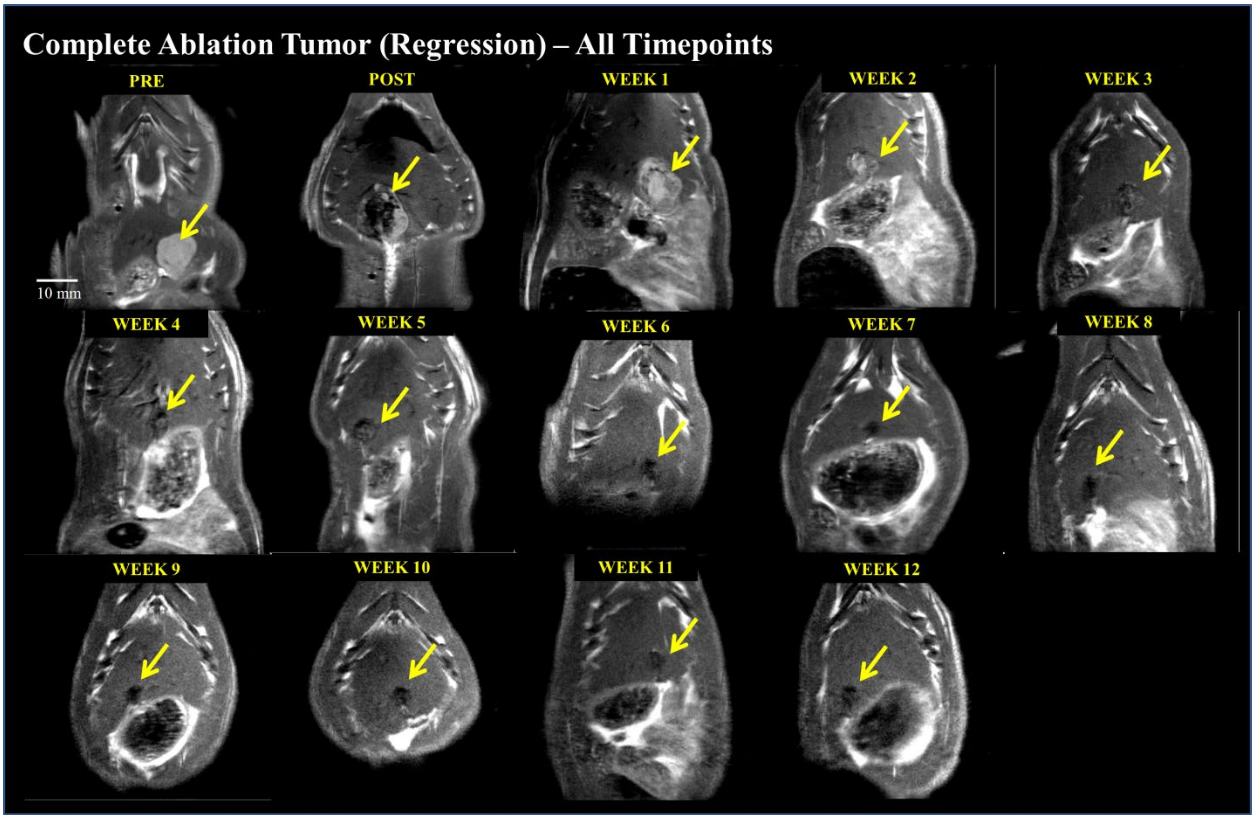

Figure S2. **T2W MRI appearance of histotripsy treated tumor (complete ablation) at all timepoints.** Complete ablation - pre-treatment (mildly hyperintense), post treatment ablation zone (hypointense), ablation zone at weeks 1-2 (mildly hyperintense), weeks 3-6 (mildly hypointense, size regression), weeks 7-12 (hypointense, size regression) with <5 mm hypointense region observed at final imaging timepoint. This figure demonstrates all imaging timepoints for the case demonstrated in Figure 2(a).

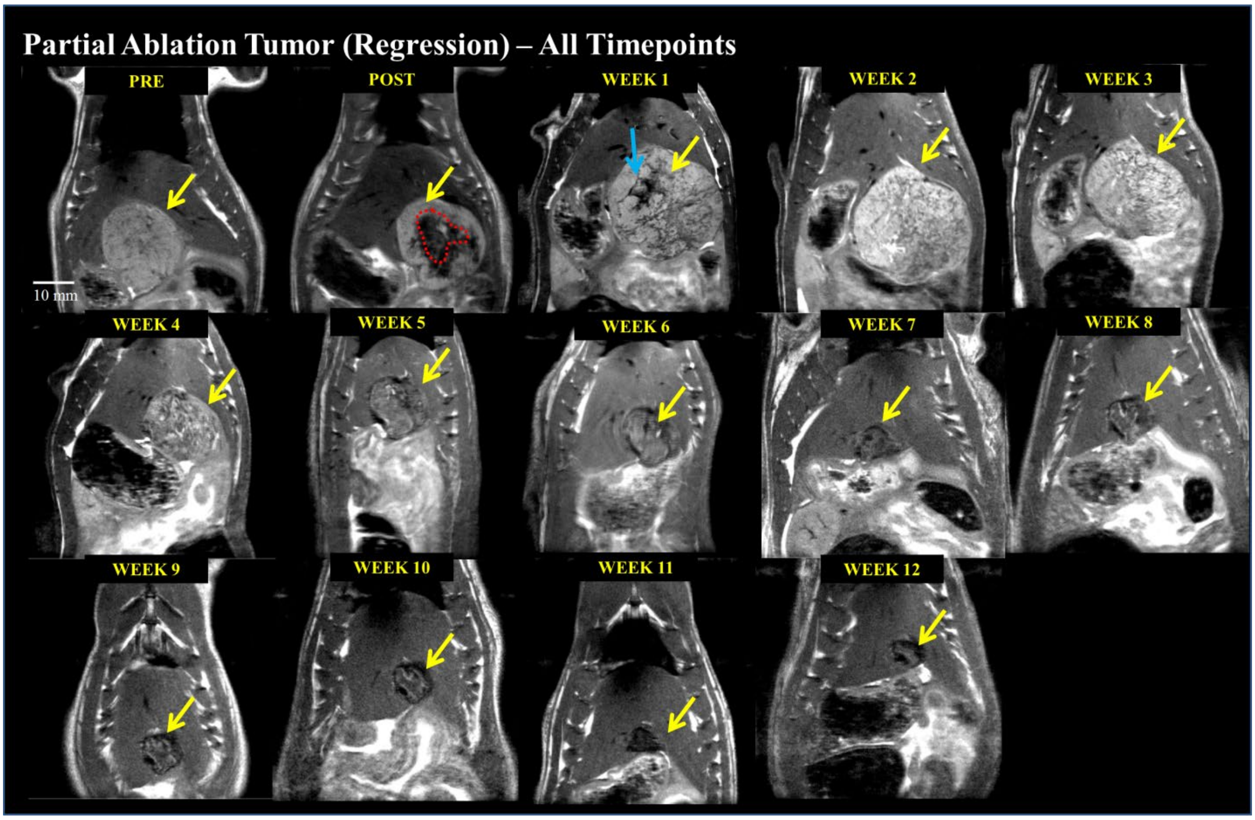

Figure S3. **T2W MRI appearance of histotripsy treated tumor (partial ablation) at all timepoints.** Partial ablation - pre-treatment (mildly hyperintense), post treatment ablation zone (hypointense, shown by red dashed lines) and untreated tumor region (mildly hyperintense), the lesion at week 1 (mildly hyperintense) with pseudoprogression characteristics (blue arrow), week 2 (mildly hyperintense), week 3 (mildly hyperintense, size regression), weeks 4-12 (gradual transformation to hypointense, size regression), and week 12 (~5 mm hypointense region). This figure demonstrates all imaging timepoints for the case demonstrated in Figure 2(b).

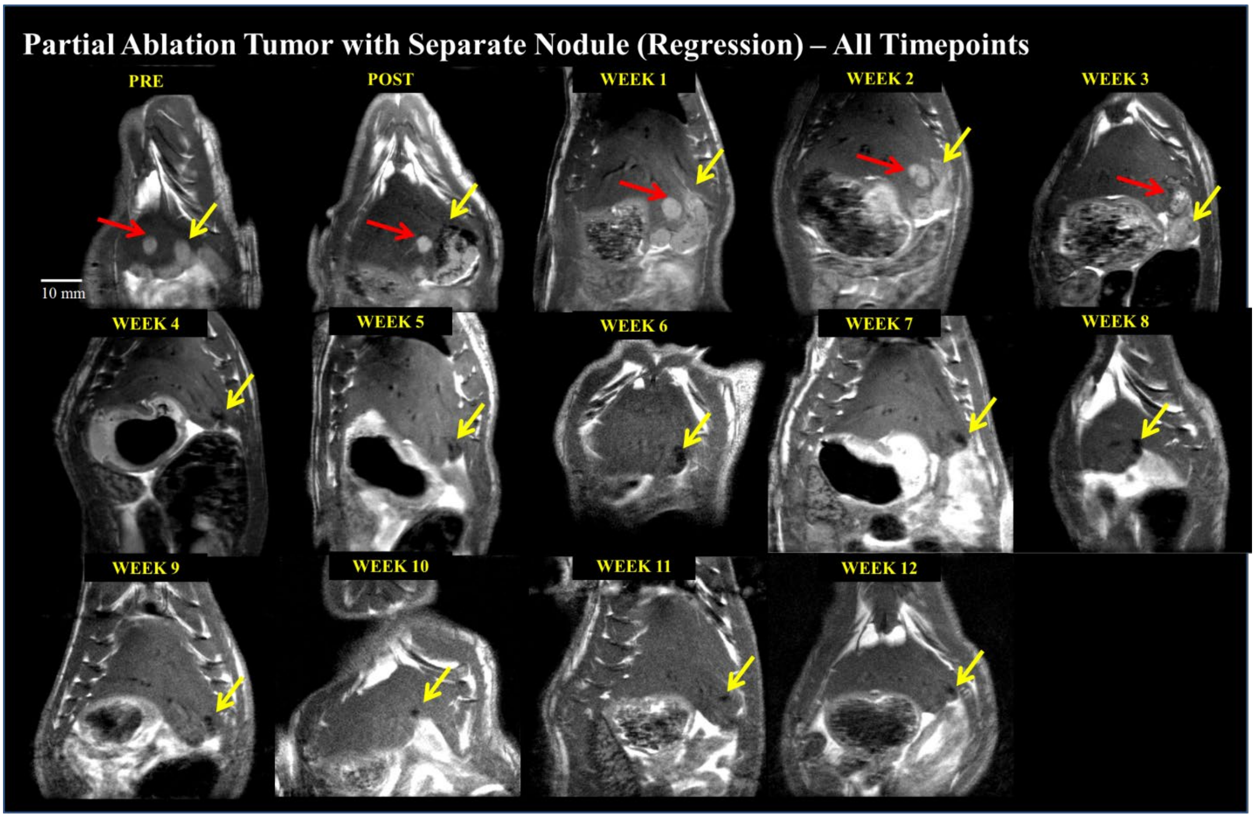

Figure S4. **T2W MRI appearance of histotripsy treated tumor (partial ablation with separate untreated nodule) at all timepoints.** Partial ablation with a separate untreated nodule (red arrow) – the lesion consisting of ablation zone and untreated nodule at weeks 1-2 (mildly hyperintense), week 3 (mild hyperintense with no size progression), weeks 4-12 (hypointense, size regression) and week 12 (<3 mm hypointense region). This figure demonstrates all imaging timepoints for the case demonstrated in Figure 2(c).

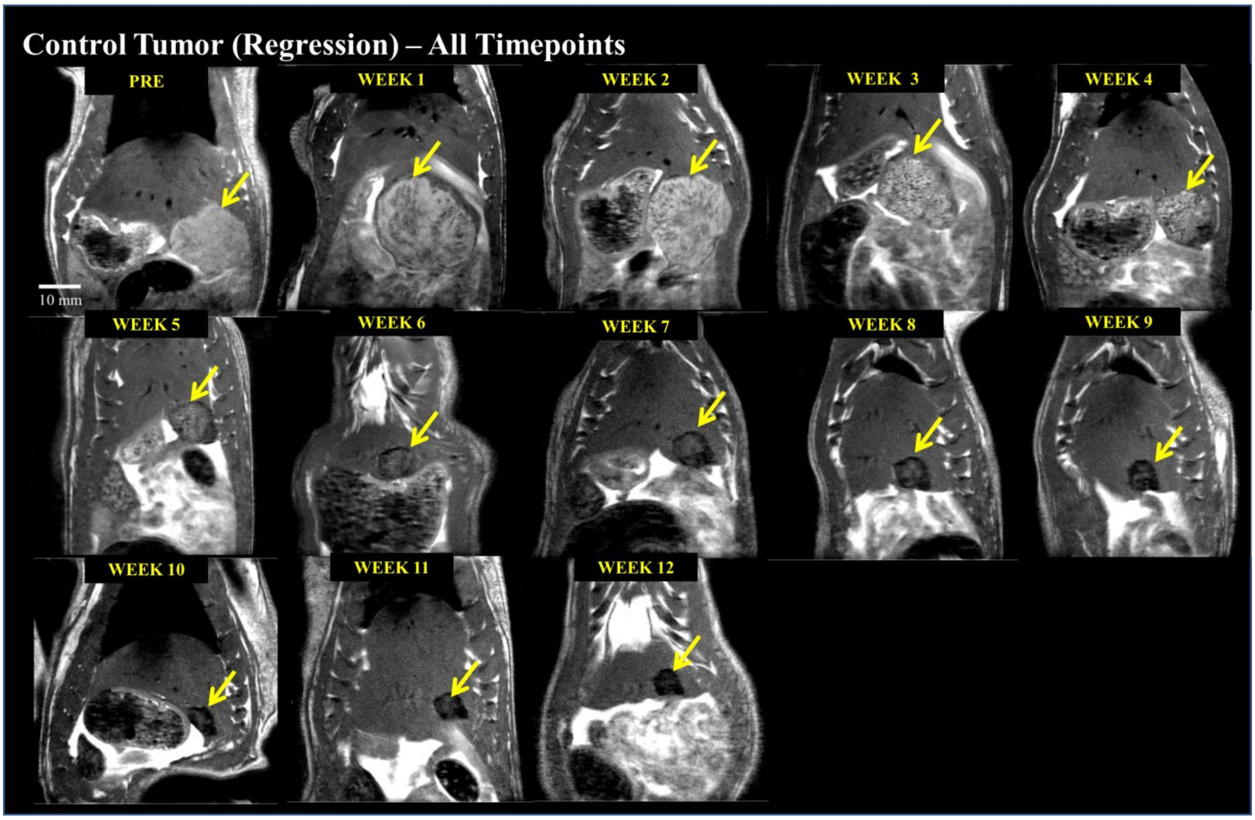

Figure S5. **T2W MRI appearance of control tumor at all timepoints.** Control tumor (yellow arrow) develops a mottled and heterogeneous appearance, similar to that of pseudoprogression by week 3, and begins to regress as evidenced by decreasing tumor burden until week 12. By week 7, the appearance of the tumor becomes hypointense and this appearance is maintained until week 12. This figure demonstrates all imaging timepoints for the case demonstrated in Figure 3(b).

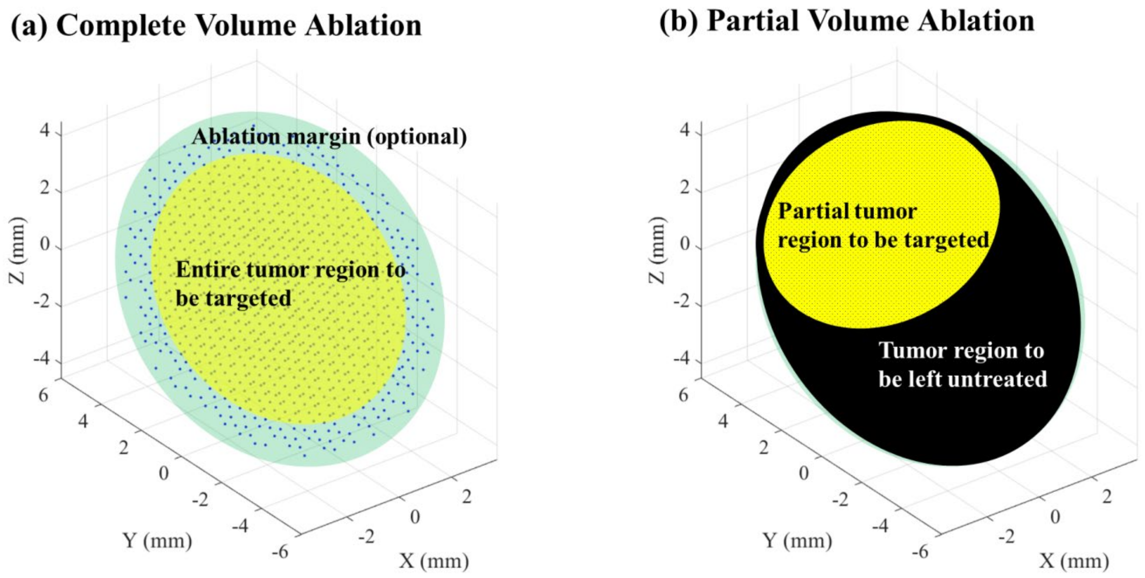

Figure S6. **Generation of treatment ellipsoid for histotripsy targeting and 3D scan path. (a)** The treatment ellipsoid (yellow) encompasses the entire 3D tumor volume to be targeted for ablation with additional margin up to 2mm (green) and contains a grid of uniformly spaced therapy focal zones (blue dots), which determines the 3D scan path. (b) For partial ablation cases, only part of the tumor volume (yellow) is targeted for ablation and the rest (black) is left untreated.
